# Electroencephalogram and Alzheimer's Disease: Clinical and Research Approaches

**DOI:** 10.1155/2014/349249

**Published:** 2014-04-24

**Authors:** Anthoula Tsolaki, Dimitrios Kazis, Ioannis Kompatsiaris, Vasiliki Kosmidou, Magda Tsolaki

**Affiliations:** ^1^Medical Physics Laboratory, Medical School, Aristotle University of Thessaloniki, 54124 Thessaloniki, Greece; ^2^3rd Department of Neurology, Aristotle University of Thessaloniki, Exochi, 57010 Thessaloniki, Greece; ^3^Centre of Research and Technology, Information Technologies Institute, 6th Klm Charilaou-Thermi Road, P.O. Box 60361, Thermi, 57001 Thessaloniki, Greece

## Abstract

Alzheimer's disease (AD) is a neurodegenerative disorder that is characterized by cognitive deficits, problems in activities of daily living, and behavioral disturbances. Electroencephalogram (EEG) has been demonstrated as a reliable tool in dementia research and diagnosis. The application of EEG in AD has a wide range of interest. EEG contributes to the differential diagnosis and the prognosis of the disease progression. Additionally such recordings can add important information related to the drug effectiveness. This review is prepared to form a knowledge platform for the project entitled “Cognitive Signal Processing Lab,” which is in progress in Information Technology Institute in Thessaloniki. The team tried to focus on the main research fields of AD via EEG and recent published studies.

## 1. Introduction

Alzheimer's disease (AD) is a neurodegenerative disorder that is characterized by cognitive deficits, disorders of activities of daily living, and behavioral disturbances. Recent research in AD is focused on defining methods to detect dementia early, preferably in the preclinical stages.

Electroencephalogram (EEG) was introduced in 1929 [1] as a method of recording human brain activity. It has been used as a tool for diagnosing AD for several decades. However, activities such as polymorphic slow waves in theta or delta range, frontal intermittent rhythmic delta activity (FIRDA), and other EEG findings in such patients are not specific and any quantification of this activity was not possible few years ago. New techniques were found to overcome these problems and in our days the hallmarks of EEG abnormalities in AD patients are as follows: a shift of the power spectrum to lower frequencies, a decrease in coherence of fast rhythms, and EEG-complexity changes, which can be found already in the early stage, in a wide frequency range. These abnormalities are thought to be associated with functional disconnections among cortical areas resulting in death of cortical neurons, axonal pathology, and cholinergic deficits [2–4].

The imaging technologies, such as computed assisted tomography and magnetic resonance imaging, allow a different approach to AD diagnosis, an in vivo view of brain structures. A few years later the development of regional metabolic methods (PET, SPECT) and the ability to map oxygen or glucose consumption and regional blood flow with functional MRI have limited the role of EEG in basic and clinical studies.

However EEG has a high sensitivity in separating AD patients from normal controls at group level and can exclude other pathologies as well. This is the reason why some clinicians suggest that all AD patients should be subjected to an EEG at least once as well as to a MRI [5, 6]. On the other hand the rate of correctly identified AD cases by electrophysiological methods varies within a wide range, between 29% and 42% in the early and between 60% and 80% in the later stages [7].

However, a new feature, the EEG spectrotemporal modulation energy may provide an automated diagnosis of AD over 91% accurate according to Trambaiolli et al. [8].

## 2. EEG and Clinical Practice

In recent years the EEG is not included in the standard diagnostic work up for AD. The diagnosis is mostly clinical, fulfilling specific criteria such as NINDS-ARDRAS and DSM [9, 10]. This diagnosis is supported by a structural imaging technique as well as blood tests. The EEG is the only diagnostic tool that shows directly the cortical neuronal functioning. However visual analysis provides only a few diagnostic clues for AD differential diagnosis. Abnormal activities in early stages of AD are not usually seen visually, so EEG cannot be used in every day clinical practice for the early diagnosis of AD, in a preclinical stage. However, an abnormal EEG in early stages can be useful excluding pseudodementia [11, 12]. In later stages of the disease abnormal findings such as slow waves are very common. A normal EEG in these patients raises questions about the diagnosis of AD making the diagnosis of subcortical dementia or frontal lobe degeneration more possible.

Moreover EEG can detect epileptic activity, a serious factor for the prognosis of the disease. At younger age, greater cognitive decline and history of antipsychotic drug use are considered to be independent risk factors for seizures in AD [13]. Seizures in patients with AD have been the subject of extensive research over the last several decades, and there are opposing opinions about their relevance to AD pathophysiology. Because of the low frequency of seizures and their late onset in AD, it was believed that they play a minor role in AD. Seizures were presented as a consequence of neurodegeneration rather than a contributing factor. However clinical data indicates that seizures can occur in earlier stages of AD as well, particularly in familial AD. This is the reason why seizures may be related to AD pathophysiology [14].

It is noteworthy that seizure frequency in patients with mild to moderate AD in clinical trials is similar to the frequency observed in longer observational cohort studies but also higher than expected in the general elderly population. Some studies suggest that seizures appear to be fairly uncommon comorbidity [15, 16], whereas other studies report a higher incidence of seizures in patients with AD [17–20].

Epileptic activity has a harmful impact on patients, especially on AD patients. Therefore greater attention is required because seizures can easily go unrecognized and untreated [21]. On the basis of the relationship between phospho-*τ* protein, cognitive decline, and epileptogenicity, Ferrazzoli et al. suggest that high liquoral phospho-*τ* levels and epileptiform EEG pattern may provide an early identification of patients with dementia and/or represent an aggressive phenotype of dementia [22].

Common clinical features of patients with amnestic mild cognitive impairment—aMCI- or AD-associated epilepsy—include early age at onset of cognitive decline, early incidence of seizures in the disease course, unilateral temporal epileptic foci detection by serial/extended EEG, transient cognitive dysfunction and good seizure control and tolerability with lamotrigine [23, 24], and levetiracetam [24]. The careful identification and treatment of epilepsy in such patients is crucial because it may improve their clinical course [24].

## 3. EEG Features in AD

Conventional visual analyses of resting stage EEG features in AD patients are characterized by an increase of widespread delta and theta activity as well as a reduction in posterior alpha and beta activity [25, 26]. As it has already been mentioned earlier, these special features appear only in late stages of AD.

Computerized EEG spectral analysis (qEEG) in AD provides more quantitative data than visual analysis. The qEEG has also shown a power increase of delta and theta power and a parallel power decrease in alpha and beta activity compared with those of normal elderly subjects. To be more precise the amount of relative theta band increases and that of the fast alpha range decreases. There is also a disrupted functional connectivity between frontoparietal and frontotemporal regions in the alpha and beta frequency bands [27]. Global coherence and global correlation dimension, which are both measures of functional connectivity, both differ within specific frequency bands pointing to a decreased functional connectivity in the alpha and in the theta frequency bands [3]. Remarkably increased omega complexity and lower synchronization likelihood are also observed in AD in the 0.5–25 Hz frequency ranges [4].

## 4. Sleep EEG in AD 

Sleep EEG can add important information in an AD patient's profile. Limitations arise when visually comparing EEG between normal elderly and mild AD patients. However, differences in sleep architecture in AD patients can be observed. Firstly, there is a reduced percentage of slow wave sleep in patients with AD [28, 29]. Secondly, these patients wake up several times during nighttime and it is usually for a prolonged time. As a result of the above, the recordings show an increased percentage of wakefulness and stage N1 (referring to the transition of the brain from alpha waves having a frequency of 8–13 Hz to theta waves having a frequency of 4–7 Hz). This stage is sometimes referred to as somnolence or drowsy sleep [30]. Consequently there is a difficulty to score the sleep EEG, due to the increased N1 stage and the decrease of sleep spindles which is the hallmark of NREM sleep, especially in stage N2 (characterized by sleep spindles ranging from 11 to 16 Hz and K-complexes). During this stage, muscular activity as measured by EMG decreases, and conscious awareness of the external environment disappears. This stage occupies 45–55% of total sleep in adults [30]. Sleep spindles are less frequent, have a shorter duration, and are not well formed in AD patients. Similar findings apply also to another sleep component, the K-complexes [31, 32].

The most interesting findings however are related to REM sleep. The amount of REM is reduced in patients with AD and this change is mostly seen in later stages of the disease [12, 33, 34]. By analyzing temporal lobe rhythms using spectral analysis, researchers classified correctly 100% of healthy controls and AD patients [35]. It is suggested that EEG slowing is more prominent in REM sleep than in the awake EEG. Hassainia et al. found that, in REM sleep, EEG slowing was greater in the temporoparietal and frontal regions, whereas during wakefulness EEG slowing was greater for the frontal pole [36].

Findings from human and animal studies are supportive of a cholinergic system dysfunction in patients with AD [37]. It is believed that REM sleep abnormalities are also indicative of this cholinergic circuit deterioration.

It seems that REM sleep EEG is a better biomarker for patients with AD than awake EEG or EEG performed during other sleep stages. However, a greater number of patients and studies are needed to confirm these findings and use the sleep EEG as a clinical tool in patients with AD.

## 5. EEG and AD Prognosis

The most important issue to investigate nowadays is whether someone will progress from a cognitive intact condition or MCI to AD. Recent scientific data suggests that specific EEG markers are correlated to the prognosis of conversion. Such markers are the increased theta/gamma ratio, the increased alpha3/alpha2 ratio, and the increase of high alpha frequency which seems to be associated with conversion to AD. Theta/gamma and alpha3/alpha2 ratio could be used as prognostic markers in MCI patients. According to recent research data, EEG markers allow a correct classification up to 88.3% [38]. The detection of this population may help us to make an earlier diagnosis and intervention.

On the other hand electroencephalographic rhythms are known to be abnormal in subjects with AD. According to recent data these sources are also sensitive to the progression of early stage AD over the course of one year. In this case EEG sources might represent cost-effective and noninvasive markers with which we will be able to detect AD patients who are expected to decline faster [39].

Researchers attempt to correlate the EEG signals not only with the general decline of an AD patient but also with specific aspects that follow this decline. An interesting example of the above is the attempt of Rodriguez et al. to use qEEG measures as prognostic markers in AD. In a preliminary study on 31 AD patients, loss of daily living activities and death were related to right delta relative power whereas the onset of incontinence was related to right theta relative power [40].

## 6. EEG and Neuropsychological Assessment 

There is correlation between the degree of the EEG abnormality and the cognitive impairment [26, 27, 41]. Recent scientific data has shown correlations between EEG delta and alpha1 activity and MMSE scores in left temporoparietal cortex. These results support the hypothesis of an asymmetrical progression of the AD [41]. Hsiao et al. found that MMSE scores were strongly correlated with the magnitudes of spectral power at the theta and alpha bands in posterior portion of default brain network [27]. Moretti et al. suggested that theta/gamma ratio of relative power at peak frequency is significantly associated with memory decline [42]. MCI to AD converters had increased alpha3/alpha2 ratios and worse performance on verbal learning tests, whereas MCI to non-AD converters had increased both theta/gamma and alpha3/alpha2 ratios and worse cognitive performance in nonverbal learning tests, abstract thinking, and letter fluency [38]. Babiloni et al. in a recent paper that correlated MRI data, cognitive test performance, and resting state EEG rhythms found that the better the score of the cognitive tests, the higher the gray matter volume and the alpha sources, and the lower the pathological delta sources [43]. The eLORETA correlation analysis indicated that lagged phase synchronization in the theta band had a negative correlation with the MMSE scores, which means that the greater cognitive decline as presented by a low MMSE score comes along with higher functional connectivity in the theta band. This included specific cortical regions such as the left temporal-right prefrontal and left anterior temporal-right central cortex. Significant correlations of theta connectivity with the MMSE scores were also observed between the left temporal-midcentral area and the right temporal-inferior parietal cortex [44].

## 7. EEG and AD Differential Diagnosis 

Despite the fact that EEG may not provide accurate information regarding the diagnosis by itself, it can provide useful data that improves the overall diagnostic accuracy as well as a distinction between the disease stages and other similar neurodegenerative disorders.

Graphical analysis of resting EEG interchannel coherence is an efficacious method for noninvasive screening for MCI and early AD [45]. On the same conclusion separating MCI from early AD patients led another study using coherence analysis of EEG signal during a cognitive task [46]. Alternative EEG signal analysis using a pair of markers (sNAT—neuronal activity topography—and vNAT) which is derived from the power spectrum drove Musha et al. to the likelihood diagram composed of sNAT and vNAT, that enables tracing the NAT state of a test subject approaching the AD area and hence detection of AD patients in the very early stage. The marker sNAT characterizes neuronal activity as hyper- or hypoactivity compared to normal controls. The other marker vNAT denotes the over- or undersynchronous collective neuronal activities as compared with normal controls [47].

Furthermore the combination of EEG data, the neuropsychological assessment, and the cardiovascular history in a logistic model increased overall dementia and MCI diagnosis accuracy from 80% to 92%. The same method was able to identify also the following subgroups (with accuracies): AD (92%; 12/13), vascular dementia (VAD) (73%; 8/11), mixed dementia (100%; 4/4), and MCI (80%; 4/5) [48].

It is noteworthy that EEG can also be useful to detect vascular cognitive impairment, no dementia (vCIND) that is a prevalent and potentially a preventable disorder [49–51].

The differential diagnosis between AD and other dementia is another field where EEG signals may help. For example, the differential diagnosis of pure AD and mixed type of dementia was another field that EEG contributed to even more than clinical symptoms and neuropsychology did. An alteration of frequency power may reflect cortical or subcortical pathology [52]. In addition, in aMCI and AD subjects resting state posterior delta and alpha EEG rhythms seem to be more sensitive to AD neurodegenerative processes and cognitive status rather than to concomitant lesions to white matter [53].

A recent review about the utility of EEG in diagnosing dementia suggests also that EEG may be useful as an adjunct in the diagnosis of DLB and AD [54]. The grand total EEG (GTE) score and the frontal intermittent rhythmic delta activity (FIRDA) can be helpful in the differential diagnosis between DLB and AD with good sensitivity and specificity [55].

Furthermore Bonanni et al., using P300, suggested that this EEG derivative technique is able to differentially diagnose Lewy body disease and AD. Particularly they showed that gradient inversion and delayed P300 responses in frontal derivations presented differences between DLB and AD patients with a sensitivity of 70% and a specificity of 97% [56, 57].

EEG is usually normal in FTD and focal changes can be seen in advanced VAD [54], especially if vascular lesions are extended [58]. Selected parameters of qEEG could also be used in addition to differential diagnosis between AD and subcortical vascular dementia with the limitation of the same level of dementia severity [59].

Finally, it is common knowledge that the typical pseudoperiodic pattern with sharp wave complexes is seen in middle and late stages of Creutzfeldt-Jakob disease [13].

## 8. EEG and AD Biomarkers

Stomrud et al. believed in the preclinical neuropathologic processes and tried to correlate the CSF biomarkers with the slowing of EEG activity in healthy elderly. Their results suggested that CSF biomarkers and EEG theta activity might indicate early abnormal degenerative changes in the brain [60]. A positive correlation between CSF Tau levels and ratio of alpha/delta global field power was found in AD patients, whereas no significant correlations between EEG slowing and CSF Tau levels were found in patients with MCI or in healthy control subjects [61].

Ferrazzoli et al. in their recent review suggest that qualitative EEG analysis integrated with cerebrospinal biomarkers may be extensively used to better define dementia. In this review the relationship between phospho-Tau protein levels and epileptiform EEG pattern was underlined [22].

A newly introduced EEG analysis named method of neuronal dysfunction (DIMENSION) found a negative correlation between the mean value of EEG alpha dipolarity and p-Tau 181 as well as the ratio p-Tau 81/A*β* 42 and a positive correlation between the standard deviation of EEG alpha dipolarity [62].

## 9. EEG and Apolipoprotein E4

Apolipoprotein E (APOE) is the major known genetic risk factor for late-onset AD. Although the relationships between APOE and *β*-amyloid are well described, the APOE effect on the brain is complex and multiple. For example, APOE appears to affect brain network activity that might be involved in the neurodegenerative pathophysiology [63]. However evidence has suggested that the influence of ApoE *ε*4 allele may commence in early life. For example, a recent study on the modulatory effects of APOE *ε*4 on regional neural activity as well as interregional neural interactions in a young population aged 19–21 using functional connectivity analyses revealed a right-lateralized network that differentiates *ε*4 carriers and noncarriers, with lower connectivity strengths for the former [64].

The APOE-brain networks relationship in cognitively healthy individuals has been widely investigated. However only few data has been reported on the effect of APOE genotype on resting state functional connectivity in AD. A significant decrease in alpha1 oscillations exhibits in parietooccipital regions of AD patients brain compared with controls. The APOE *ε*4 allele carriers had reduced alpha1 activity in the left inferior parietal and tempooccipital cortex relative to noncarriers. When analyzing carriers versus noncarriers among patients with early AD, decreased alpha2 lagged phase synchronization (a measure of physiological nonlinear connectivity) was found between lateral frontal areas and parietotemporal areas across hemispheres. It is noteworthy that although APOE *ε*4 is a genetic risk factor for AD, it appears to have a negative impact on cortical rhythms and functional connectivity even after the development of the disease [44]. AD patients *ε*4 carriers have more pronounced slow wave activity than AD patients *ε*4 noncarriers, although the progression rate does not change. These differences in EEG may suggest differences in the degree of the cholinergic deficit in these subgroups [65].

The opposite opinion was presented by an older study of coherence, suggesting that APOE *ε*4 does not influence the slowing down of EEG rhythms. Researchers do agree however on the APOE effect on functional connectivity as assessed by EEG coherence [66].

Comparing APOE *ε*4 carriers and noncarriers AD patients, on a visual evoked potentials study, revealed also that *ε*4 carriers had significantly longer peak latencies (the latencies of the typical peaks of the VEP responses, N75, P100, N135, and P180) and a trend to higher interpeak latencies of late potential components. These data lead to the conclusion that the APOE *ε*4 allele promotes neuronal dysfunction [67].

Neuroimaging shows brain-functional differences due to APOE polymorphisms and these differences may exist decades before the increased risk period for AD [68]. In addition to the above a recent study comes to support that qEEG can differentiate AD in very early stages and that it can be even accurate if the genetic profile of APOE *ε*4 is combined [69].

The effect of the genetic profile on the EEG rhythms is a great research subject but is not the only. Recent studies on the special features of *ε*4 carriers managed to differentiate APOE *ε*4 carriers from noncarriers, using olfactory ERPs in an odor/visual congruency task [70, 71].

## 10. EEG and Pharmaceutical Intervention in AD

Cholinesterase inhibitors (ChEIs) are the most widely used symptomatic treatment for mild to severe AD patients, while N-methyl-d-aspartic acid (NMDA) receptor antagonist memantine is licensed for use in moderate to severe AD patients. Several studies have shown that ChEIs affect both resting state EEG rhythms and cognitive functions in AD patients [72].

Increased EEG slow wave activity in patients with AD may reflect the cholinergic deficit. According to this hypothesis, effective ChEIs treatment should be leading to a decrease in EEG slow wave activity. Several studies have been done before and after the beginning of medication with ChEIs. Adler et al. only a week after the initiation of rivastigmine (immediate release rivastigmine) noticed that theta power decreased significantly. According to this study the treatment responders had a greater decrease in theta power after one week of treatment and a better short-term memory at baseline than nonresponders (therapeutic efficacy was determined six months after treatment initiation). This data suggests that EEG can not only detect the therapeutic effect of the drugs but also be used, combined with the neuropsychological assessment, for predicting response to rivastigmine in patients with Alzheimer's disease [73].

Babiloni et al. studied mild AD patients before treatment with donepezil and found that posterior sources of delta, alpha1, and alpha2 frequencies were greater in amplitude in nonresponders. A year later after the initiation of treatment, a lesser magnitude reduction of occipital and temporal alpha1 sources characterized responders. These results suggest that responders and nonresponders had different EEG cortical rhythms [74].

Gianotti et al. found that after a three-month period of rivastigmine input the spectral analysis of the EEG data showed a significant power decrease in the delta and theta frequency bands in frontal, parietal, and temporal regions. A correlation analysis of the differences between the cognitive performances and the LORETA-computed intracortical activity also showed in the *α*1 frequency band better cognitive performance with increased cortical activity in the left insula. These data suggests a shift of the power spectrum towards normalization [75]. A previous study of Rodriguez et al. on donepezil effect concluded with the observation that metabolic activation of ChEIs might especially influence posterior parietal region, which is often affected by hypoperfusion in AD [76]. The qEEG may be useful for measuring AD treatment responses. It can also monitor treatment of AD (accurately reflecting treatment in a dose dependent manner). These results were independent of the specific medication monitored, galantamine, memantine, nicotine, and rivastigmine [77].

Last but not least Mizuno et al. observed the effects of donepezil on both the cognitive function and sleep patterns of AD patients. The increase of EEG slow wave activity in AD patients, which is more prominent during REM sleep, probably reflects a cholinergic deficit. The authors found that the percentage of REM sleep to total sleep time increased after the administration of donepezil. This increase may contribute to the improvement of cognitive function in AD patients treated with donepezil [78]. Taking all the above into account we could say that qEEG may be useful for measuring AD treatment responses.

## 11. EEG and Other Imaging Techniques

EEG signals have been correlated with the outcomes of other neuroimaging techniques. In mild to moderate AD patients, it has been shown that hippocampal volumes are 27% smaller than in normal elderly controls, whereas patients with MCI show a volume reduction of 11% [79]. AD is associated also with neuronal loss in the thalamus and basal ganglia. Main sites of degeneration in AD include anterodorsal, centromedial, and pulvinar nuclei [80]. Moretti et al. in their recent study found that MCI subjects with higher a3/a2 ratios when compared with subjects with lower and middle a3/a2 ratios showed minor atrophy in the ventral stream of basal ganglia (head of caudate nuclei and accumbens nuclei bilaterally) and of the pulvinar nuclei in the thalamus [81].

A recent study has found a correlation between the brain electrical activity and discrete-mapped hippocampal areas in subjects with AD. The results of this study show that in AD patients the increase of both alpha3 rhythm spectral power and alpha3/alpha2 power ratio is correlated with the decrease of left hippocampal gray matter volumes. This outcome could suggest that there is compensatory synchronization in high alpha rhythm in an effort to balance the degenerative process or that the disruption of the order of a stable attractor network prevents the synchronization of large neural assemblies, inducing an increase of high alpha power [82]. Amygdala-hippocampal complex (AHC) atrophy is associated with memory deficits as well as with the increase of theta/gamma and alpha3/alpha2 ratio. Recent data showed that the increase of theta/gamma ratio is best associated with amygdalar atrophy whereas alpha3/alpha2 ratio is best associated with hippocampal atrophy [83]. Additional to the AHC atrophy, cerebrovascular (CV) damage which is considered to be another risk factor for dementia was associated with EEG relative power, separately computed for delta, theta, alpha1, alpha2, and alpha3 frequency bands. The result of this analysis showed in the spectral band power that the severity of cerebrovascular damage was associated with increased delta power and decreased alpha2 power. In addition the theta/alpha1 ratio could be a reliable index for the estimation of the individual extent of CV damage [84].

Babiloni et al. in their recent work suggested the hypothesis that, in amnesic MCI and AD subjects, abnormalities of EEG rhythms exist due to the cortical atrophy across the disease. Their results showed that abnormalities of resting state cortical EEG rhythms are strictly related to neurodegeneration and cognition [43].

Finally the new method of EEG analysis, DIMENSION, correlated EEG recordings with data of single-photon emission computed tomography. According to their results AD patients with parietal hypoperfusion had decreasing mean value of EEG alpha dipolarity and increasing standard deviation of EEG alpha dipolarity [62].

## 12. Discussion

EEG has been demonstrated as a reliable diagnostic tool in dementia research [2, 85, 86]. Intensive research has been performed on the EEG especially in AD. AD is a cortical dementia in which EEG rhythms abnormalities are more frequently shown, whereas subcortical dementia exhibits relatively normal EEG patterns. The coherence analysis of EEG in AD also allows noninvasive assessment of synaptic dysfunction. In a few words EEG abnormalities reflect anatomical and functional deficits of the cerebral cortex in AD.

Using EEG with contemporary statistical methods seems to be a reliable method to classify the clinical cases of cognitive impairment, although when the comorbidity is high this classification is not so well effective [87].

It is noteworthy that slow waves over the temporal areas, which usually characterize AD, are occasionally seen in the EEG of normal elderly subjects. However the main features of these “nonpathological” slow waves ((1) they do not disrupt background activity, (2) they are not associated with a substantial asymmetry of the alpha rhythm, (3) their morphology is usually rounded and their voltage is usually greater than 60–70 *μ*V, (4) they are attenuated by mental activity and eye opening and their prevalence is increased by drowsiness and hyperventilation and finally, (5) they occur sporadically as single waves or in pairs, not in longer rhythmic trains) can differentiate them from the AD correlated slow activity [88].

As far as antidementia medication is mentioned, EEG might help monitoring. Increased EEG slow wave activity in patients with AD, as already mentioned, probably reflects a cholinergic deficit. Based on this hypothesis, consequently an effective cholinesterase inhibitor treatment should lead to a decrease in EEG slow wave activity. This effect of cholinesterase inhibitors treatment has been frequently observed as it is underlined in the recent review by Babiloni et al. [72]. Unfortunately in clinical practice only half of the patients treated with cholinesterase inhibitors show a visible improvement in cognitive performance. On the other hand cholinesterase inhibitor treatment is associated with various side effects and high cost [73]. That is why it is important to detect and select the patients that respond to the treatment and EEG may help us according to Babiloni et al. [74].

The genetic profile of APOE *ε*4 is associated with selective decrease in functional connectivity, which indicates a connection to AD pathogenetic mechanisms. Further evidence of a possible pathogenetic role of APOE *ε*4 is its presence in the neuropathological lesions, in senile plaques, neurofibrillary tangles, and cerebrovascular amyloid, which are the hallmarks of AD [89].

Taking into account all the recent scientific results and all the current EEG research in AD, we agree with previous statements which support that, despite the evidence of abnormal cortical rhythms in MCI and AD, EEG analysis alone is unable to allow a diagnosis of the disease. Additional biological parameters are needed for this purpose [85]. The main EEG application should be the differential diagnosis between dementia and other conditions characterized by peculiar EEG patterns such as Creutzfeldt-Jacob's disease, toxic-metabolic encephalopathy, or other psychiatric condition such as pseudodepressive dementia [90]. In a broader sense EEG can also be used to stage the severity of dementia and provide information for prognostic purposes [91]. Finally, it would be useful in evaluating the biological effect of drugs [72, 92].

In conclusion, we believe that the biological complexity of the brain function and the physical “sum” effect of brain electrical fields on EEG recordings make the understanding of EEG signals a very difficult task.

## Abbreviations

AD: Alzheimer's disease
VAD: Vascular dementia
FTD: Frontotemporal dementia
LBD: Lewy body disease
MCI: Mild cognitive impairment
MMSE: Minimental state examination
REM: Rapid eye movement
NREM: Nonrapid eye movement
NAT: Neuronal activity topography
APOE: Apolipoprotein E.
